# Variation in dermcidin expression in a range of primary human tumours and in hypoxic/oxidatively stressed human cell lines

**DOI:** 10.1038/sj.bjc.6604458

**Published:** 2008-07-01

**Authors:** G D Stewart, R J E Skipworth, C J Pennington, A G Lowrie, D A C Deans, D R Edwards, F K Habib, A C P Riddick, K C H Fearon, J A Ross

**Affiliations:** 1 Tissue Injury and Repair Group, Clinical and Surgical Sciences (Surgery), University of Edinburgh, Edinburgh EH16 4SB, UK; 2 Prostate Research Group, Western General Hospital, Edinburgh EH4 2XU, UK; 3 School of Biological Sciences, University of East Anglia, Norwich NR4 7TJ, UK

**Keywords:** dermcidin, prostate cancer, pancreatic cancer, gastric cancer, oesophageal cancer

## Abstract

Dermcidin acts as a survival factor in a variety of cancer cell lines under hypoxia or oxidative stress. The aim of this study was to evaluate dermcidin expression in cell lines following simulation of tumour microenvironmental conditions and in a range of primary tumours. Tumour tissues were collected from patients with oesophageal (28 samples), gastric (20), pancreatic (five), bile duct (one) and prostatic (52) carcinomas as well as 30 benign tissue samples, for assessment of dermcidin mRNA levels using real-time PCR. Dermcidin expression was assessed in prostatic and pancreatic cancer cell lines, with and without induction of hypoxia or oxidative stress. Dermcidin mRNA expression was very low or absent in both unstressed and stressed prostate cell lines. None of the primary prostate tissue, benign or malignant, expressed dermcidin mRNA. Only two (4%) of the gastro-oesophageal cancer samples expressed moderate quantities of dermcidin mRNA. However, three (60%) of the pancreatic cancer samples and the single cholangiocarcinoma specimen had moderate/high levels of dermcidin expression. Of the two pancreatic cancer cell lines, one expressed dermcidin moderately but neither showed a response to hypoxia or oxidative stress. Expression of dermcidin in human primary tumours appears highly variable and is not induced substantially by hypoxia/oxidative stress in cell line model systems. The relationship of these findings to dermcidin protein levels and cell survival remains to be determined.

We have previously documented that dermcidin (DCD) acts as a survival factor in prostate cancer cells exposed to either hypoxia or oxidative stress (Stewart *et al*, 2007), and hepatoma cell lines subject to oxidative stress (Lowrie *et al*, 2006). Porter *et al* (2003) determined that DCD expression in breast cancer cell lines was associated with cancer cell growth and survival . As such, *DCD* has been suggested as a candidate oncogene in breast cancer (Porter *et al*, 2003). Cunningham *et al* (1998) studied the role of oxidative stress in neuronal degeneration and identified a 30 amino acid survival-promoting peptide, which they named Y-P30. The Y-P30 peptide was present in medium conditioned by human retinoblastoma cells and a mouse hippocampal cell line exposed to hydrogen peroxide. Y-P30 was subsequently identified as comprising part of a 110 amino acid polypeptide which Cunningham *et al*, (2002) named diffusible survival evasion peptide (DSEP). DSEP-overexpressing neuronal cells were also found to have an increased resistance to oxidative stress (Cunningham *et al*, 2002).

The *DCD* gene was identified on chromosome 12q13.1 and encodes different proteins with divergent biological functions (Schittek *et al*, 2001; Stewart *et al*, 2008). The protein products of the 110 amino acid DCD polypeptide (Figure 1) include the 47 amino acid DCD-1 peptide (a skin antimicrobial) (Schittek *et al*, 2001) and the 30 amino acid proteolysis-inducing factor-core peptide (PIF-CP) (Lowrie *et al*, 2006), the latter of which is identical to Y-P30 (Cunningham *et al*, 1998). The DCD protein (Schittek *et al*, 2001) is identical to human cachexia-associated protein (HCAP) (Akerblom and Murry, 1998) and DSEP (Cunningham *et al*, 2002). Furthermore, when glycosylated, PIF is reported to behave as a factor in cancer cachexia (Todorov *et al*, 1996). Control of expression and post-translational processing of the various protein subunits of DCD has not yet been fully elucidated (Figure 1).

As DCD appears to encode both putative tumour survival and cachectic factors, it represents an important potential therapeutic target in cancer patients. Thus, this study aimed to evaluate the expression of DCD mRNA (as a surrogate of Y-P30/PIF-CP expression) using quantitative real-time PCR in a range of primary tumours. Specifically, DCD expression by primary tumours from patients with gastro-oesophageal, pancreatic, bile duct, and prostate cancer was analysed. Furthermore, DCD expression was also assessed in normal or benign tissue from the prostate, stomach, oesophagus, and pancreas. Prostate cancer represents the first tumour in which DCD/HCAP was described whereas the upper gastrointestinal cancer types are associated significantly with the development of cancer cachexia (DeWys *et al*, 1980). Finally, to determine if DCD expression is upregulated in response to stressors found in the cancer microenvironment (Brown and Wilson, 2004; Valko *et al*, 2006; Vaupel *et al*, 2007), we have analysed changes in the expression of DCD mRNA in prostate and pancreatic cell lines *in vitro* in response to hypoxia or oxidative stress.

## Materials and methods

### Cell lines

The following human cancer cell lines were used in these studies: hormone-sensitive prostate cancer cell line LNCaP; hormone-insensitive prostate cancer cell lines PC-3, DU145 (all obtained from the European Collection of Cell Cultures, Porton Down, UK (ECACC)) and PC-3M (kindly donated by Dr C Pettaway, University of Texas, MD Anderson Cancer Center, Houston, TX, USA); and pancreatic adenocarcinoma cell lines CFPAC (ECACC) and MIA-Pa-Ca-2 (ECACC). Two or more cell lines were utilised for each organ site to obtain corroborative results.

The prostate cell lines were cultured in RPMI-1640 medium (Invitrogen, Paisley, UK). Pancreatic cancer cell lines were cultured in Dulbecco's modified Eagle's medium (Invitrogen). Media were supplemented with 10% fetal calf serum (FCS), 50 units ml^−1^ penicillin, 50 *μ*g ml^−1^ streptomycin and 2 mmol glutamine (all Invitrogen). Cells were grown in 75 cm^2^ flasks (Corning BV, Schipol-Rijk, The Netherlands).

### Transfection of cells with DCD cDNA for use as positive control

A robust positive control for real-time PCR was created by stably transfecting PC-3M cells with a DCD-expressing vector. Wang *et al* (2003) demonstrated that the PC-3M cell line does not express DCD mRNA and it was thus chosen as a suitable cell line to be transfected with a pcDNA3.1+DCD vector. The pcDNA3.1+ mammalian expression vector (Invitrogen) had full length DCD cDNA directionally cloned into the multiple cloning site using the *Eco*R-I and *Bam*H-I restriction enzymes (Promega, Southampton, UK). pcDNA3.1+DCD vector was stably transfected into PC-3M cells using FuGENE-6 (Roche Applied Science, Lewes, UK). Geneticin selection antibiotic (Invitrogen) (600 *μ*g ml^−1^) was used to select transfected cells and eventually create stable transfectants. Sham transfected PC-3M cells were created using an empty pcDNA3.1 vector as a control. A total of 300 *μ*g ml^−1^ Geneticin was used to supplement medium of transfected cells to maintain the transfected cells.

### Induction of oxidative stress in cancer cell lines

Stock solutions of 30% hydrogen peroxide, menadione sodium bisulphite and glucose/glucose oxidase (GO) (Sigma, Poole, UK) were diluted in medium appropriate for the cell line to be stressed. Three different oxidative stress-inducing compounds were employed to allow confirmation that oxidative stress, rather than other mechanisms of the ROS generating agents were at play. Dose–response experiments were perfomed to determine the approptiate doses of the oxidative stress-inducing agents to stress the cell lines but not induce global cell necrosis. To assess levels of cell death following oxidative stress induction, cells were harvested, washed and stained with propidium iodide as described previously (Stewart *et al*, 2007). One millimole of hydrogen peroxide, 50 *μ*M menadione or 5 mU ml^−1^ GO were chosen to oxidatively stress the cancer cells, resulting in mean levels of cell necrosis of 56.5, 62.5 and 30.9% respectively after 24 h exposure. Cells were incubated with oxidative stress-inducing compounds for 24 h prior to RNA isolation.

### Hypoxia induction

To simulate the hypoxic conditions within a solid tumour *in vivo*, cells were incubated for 48 h within a humidified hypoxia incubator at 0.2% oxygen (pO_2_∼1.5 mm Hg) using a PROOX 110 oxygen controller (BioSpherix Ltd, NY, USA) (Brown and Wilson, 2004; Vaupel *et al*, 2007). This level of hypoxia resulted in a mean of 62.7% cell death after 48 h. RNA was then isolated and assessed for DCD expression.

### Clinical tissue samples

#### Prostate samples

Samples of malignant and benign human prostate tissue were obtained from the Partners in Cancer Research Tissue Bank, held in the Department of Histopathology at the Norfolk and Norwich University Hospital. Details of ethical approval, procedures for obtaining informed patient consent, tissue acquisition, and histopathological and molecular quality control and validation have already been described (Riddick *et al*, 2003). Samples of malignant prostate tissue were collected from patients undergoing radical prostatectomy or channel transurethral resection of the prostate, and non-malignant samples were obtained from patients undergoing radical cystoprostatectomy for transitional cell carcinoma of the bladder or transurethral resection of the prostate for benign prostatic hyperplasia. In all 68 primary prostate specimens were obtained (Table 1). The prostate samples were made up of: 16 benign prostatic hyperplasia specimens, eight Gleason 6 samples, 31 Gleason 7 samples, three Gleason 8 samples, six Gleason 9 samples and four Gleason 10 samples.

#### Oesophageal, gastric, and pancreatic cancer samples

Patients with a firm radiological or histological diagnosis of pancreatic or gastro-oesophageal cancer were recruited from the surgical unit of the Royal Infirmary of Edinburgh. Patients provided written informed consent and the study received ethical permission from the Lothian Research Ethics Committee. Tissue was obtained from patients at the time of operation. For patients undergoing surgical resection with curative intent, a section of tumour tissue was dissected from fresh surgical specimens under the guidance of a consultant pathologist. For patients undergoing palliative bypasses for pancreatic cancer, core biopsies of tumour tissue were taken intraoperatively. Control gastric and oesophageal biopsies were collected from healthy volunteers undergoing endoscopic investigation of gastrointestinal symptoms. A consultant pathologist analysed tissue sections to confirm the presence of malignant cells in the tumour samples and the absence of malignant cells within the benign samples. Tissue samples were snap-frozen in liquid nitrogen before storage at −80°C until further analysis. Clinical information was collected prospectively.

Tissue samples were obtained from 28 oesophageal cancers and 20 gastric cancers (Table 1). Additionally, there were 10 benign control samples from patients having upper GI endoscopy for investigation of non-neoplastic pathologies.

Eleven tissue samples from patients having resections of pancreatic masses were collected. These samples included a range of diseases (Table 1): pancreatic adenocarcinoma (five samples); chronic pancreatitis (two samples); and one sample each of cholangiocarcinoma, liposarcoma, adenoma, and cystadenoma.

### RNA isolation

Total RNA was extracted from cell lines using the TRIzol method according to the manufacturer's instructions (Invitrogen). The resulting total RNA was quantified using an Ultraspec 2000 spectrophotometer (Pharmacia Biotech, Cambridge, UK).

Total RNA from the prostate tissues was isolated by first homogenising tissues in RNAzol (Biogenesis, Poole, UK) and then by using the Promega SV Total RNA Isolation System (Promega) to remove DNA and purify the RNA. RNA was resuspended in nuclease-free water and concentrations determined using a Nanodrop spectrophotometer (LabTech International, Ringmoor, UK).

For RNA preparation from intact gastro-oesophageal and pancreatic tissue samples a rotor–stator homogeniser was used for homogenisation. RNA was then extracted using an RNeasy kit (Qiagen, Crawley, UK) according to the manufacturer's instructions.

### Real-time PCR

cDNA preparation and real-time PCR were performed as previously described (Nuttall *et al*, 2003; Riddick *et al*, 2005). Briefly, 1 *μ*g of total RNA was reverse transcribed using 2 *μ*g random hexamers (Amersham, Little Chalfont, UK) and Superscript II reverse transcriptase (Life Technologies, Paisley, UK) according to the supplier's instructions. cDNA was stored at −20°C until used in the PCR.

For PCRs, specific primers and fluorogenic probes for DCD were designed using Primer Express 1.0 software (Applied Biosystems, Warrington, UK) and synthesised by Applied Biosystems. Figure 2 shows the primer and probes targets on the DCD nucleotide sequence which partially overlaps with the nucleotide sequence for the PIF-core peptide:

DCD real-time forward primer: CAAAAGGAAAATGCAGGTGAAGA

DCD real-time reverse primer: TGGAAAAAGGCCTAGACGGAG

DCD real-time probe: FAM-ACAGGCACCAAAGCCAAGGAAGCA-TAMRA

To control against amplification of genomic DNA, primers were designed to span exon boundaries to control for genomic amplification. The 18S rRNA gene was used as an endogenous control to normalise for differences in the amount of total RNA in each sample. 18S rRNA primers and probe were purchased from Applied Biosystems. PCRs were performed using the ABI Prism 7500 fast Sequence Detection System (Applied Biosystems), using the manufacturer's protocol. Each reaction was performed in 25 *μ*l and contained the equivalent of 5 ng of reverse transcribed RNA (1 ng RNA for the 18S analyses), 50% TaqMan 2 × PCR Master Mix (Applied Biosystems), 200 nM each of the forward and reverse primer, and 100 nM of probe. Conditions for the PCR were 2 min at 50°C, 10 min at 95°C and then 40 cycles, each consisting of 15 s at 95°C, and 1 min at 60°C. The ABI Prism 7500 measured the cycle–cycle changes in fluorescence in each sample and generated a kinetic profile of DNA amplification over the 40-cycle PCR. The cycle number (termed cycle threshold, or *C*_T_) at which amplification entered the exponential phase was determined and this number was used as an indicator of the amount of target RNA in each tissue, that is, a lower *C*_T_ indicated a higher quantity of starting RNA. Relative standard curves for *C*_T_ *vs* input RNA were prepared, and relative levels of starting RNA in each sample were determined. The relative standard curve method was used to determine the fold change in gene expression between treated and untreated cells. Only DNA samples with an 18S *C*_T_ value within 1.5 *C*_T_s of the median *C*_T_ for 18S were used for analysis as this value suggested that the RNA was of sufficient quality for analysis of DCD expression. We used the *C*_T_ value of DCD to classify its expression as: very high (*C*_T_<25.5), high (⩾25.5 *C*_T_<30.5), moderate (⩾30.5 *C*_T_<35.5), low/absent (⩾35.5 *C*_T_<40), or not detected/below the limits of detection (*C*_T_=40) (Nuttall *et al*, 2003). Owing to a drop off in sensitivity of the instrument, *C*_T_ values ⩾35.5 are unreliable, in terms of exact levels of mRNA expression or assessing changes in *C*_T_ values.

The DCD-transfected PC-3M cells transfected with the pcDNA3.1+DCD plasmid gave a *C*_T_ value of 23.1. Thus, the positive control was in the very high range of DCD mRNA expression.

### Data analysis

All *in vitro* experiments were performed in sextuplet and repeated three times. Where appropriate, values were expressed as means.

## Results

### Dermcidin expression levels in prostate cancer cell lines and clinical tissue samples

#### Prostate cancer cell lines with or without induction of oxidative stress/hypoxia

Prostate cancer cell lines demonstrated low or absent levels of DCD expression. PC-3 had a *C*_T_ value of 36.7, LNCaP a *C*_T_ of 39.2 and DU145 a *C*_T_ of 39.6. PC-3M had a *C*_T_ value of 40.

Each of the prostate cancer cell lines was stressed with a variety of oxidative stress-inducing agents or incubation under hypoxic conditions. Dermcidin mRNA expression was marginally altered in LNCaP cells when exposed to GO (2.4-fold increase ±1.7) or menadione (2.3-fold increase ±1.2), in DU145 cells when exposed to 0.2% hypoxia (1.7-fold increase ±1.1), and increased in PC-3M cells following hypoxia and treatment with all three oxidative stress-inducing compounds. Dermcidin expression by the PC-3 cell line was not elevated by any of the forms of stress utilised.

In all cases DCD expression failed to reach a level of expression above an unreliable low/absent level (*C*_T_ value ⩾35.5) following exposure to oxidative stress or hypoxia.

#### Primary prostate specimens

None of the 68 primary prostate samples, either benign or malignant, expressed dermcidin, all having *C*_T_-values of 40 (Table 1).

### Dermcidin expression levels in oesophageal and gastric specimens

Table 1 details the DCD expression level of the gastric and oesophageal samples. Nine (19%) of the 48 gastro-oesophageal tumour samples displayed detectable levels of DCD expression (*C*_T_<40), including four (14%) of the oesophageal cancers and five (25%) of the gastric cancers. Only two (4%) of the cancer RNA samples produced a DCD *C*_T_ value <35.5, representing reliable moderate levels of DCD expression (two gastric adenocarcinomas with *C*_T_ values of 30.5 and 31.7 respectively). None of the 10 benign control samples demonstrated detectable levels of DCD mRNA.

### Dermcidin expression levels of pancreatic cell lines and resection specimens

#### Pancreatic cancer cell lines with or without induction of oxidative stress/hypoxia

Both pancreatic cancer cell lines used in this study demonstrated DCD mRNA expression. MIA-Pa-Ca-2 and CFPAC cell lines had *C*_T_ values of 35.1 and 37.6, moderate and low/absent levels of DCD mRNA expression respectively.

Both MIA-Pa-Ca-2 and CFPAC cell lines were subjected to three oxidative stress-inducing agents or incubation under hypoxic conditions. DCD mRNA expression was marginally altered in CFPAC cells when exposed to 0.2% oxygen (2.0-fold increase ±4.1), but not following oxidative stress. There was no upregulation of DCD expression by MIA-Pa-Ca-2 on exposure to hypoxia or oxidative stress. Although, DCD expression by CFPAC cells was marginally altered following hypoxia it failed to reach a reliable, moderate level of expression.

#### Primary pancreatic mass resection samples

The histopathology and *C*_T_ values of samples that expressed DCD mRNA are shown in Table 2. Five samples (45.5%) expressed DCD mRNA, three of which had *C*_T_ values 25.5–30.5 defined as high levels of expression (Table 1): 2 metastatic pancreatic adenocarcinomas and one poorly differentiated cholangiocarcinoma. One sample, a liposarcoma, had an unreliable low/absent level of DCD expression. Overall, three of the five pancreatic cancer RNA samples (60%) had high or moderate levels of DCD expression.

## Discussion

The results of this study showed a wide range of DCD expression between different primary cancer types, namely: prostate, gastric, oesophageal, pancreatic, and bile duct cancer. DCD mRNA expression was undetectable in the primary prostate cancer samples. In contrast, 60% of primary pancreatic cancer samples expressed significant quantities of DCD mRNA. Additionally, the MIA-Pa-Ca-2 pancreatic cancer cell line was the only cell line reliably expressing DCD mRNA. However, dermcidin expression by prostatic or pancreatic cancer cell lines was not substantially increased by induction of hypoxia or oxidative stress.

The present study included a large number of patient samples from five tumour types, plus benign tissue from some of the affected organs. However, there were no samples taken from distant metastatic deposits, which have been shown to express high levels of DCD in previous studies (Wang *et al*, 2003).

In this study absolute *C*_T_ values were used to assess DCD mRNA expression. Owing to a drop off in the sensitivity of the instrument, *C*_T_ values greater than 35.5 are unreliable in terms of the amount of mRNA expressed. Furthermore, the comparison of *C*_T_ values ⩾35.5 is not reliable as differences in *C*_T_ may not represent real or meaningful differences in mRNA present. In primary tumour samples, which have a large degree of cellular heterogeneity, *C*_T_ values greater than 35.5 mRNA represent low expression. Such *C*_T_ values may reflect generally low expression or there may be a subset of cells expressing an appreciable level of the mRNA. However, in cell lines, where heterogeneity is not such an issue, a *C*_T_ value greater than 35.5 means that mRNA may be expressed (or could be absent), but if it is expressed it is at a very low level on a per cell basis.

The results from primary prostate tissue suggest that DCD was not expressed by primary prostate cancer. Moreover, if DCD was present in the immortalised prostate cancer cell lines used, it was present at a very low level. Although cancer cell lines would be expected to have a uniform cell population, heterogeneity may exist. As such, it could be speculated that there may be low or absent pan-cellular DCD expression with a small subset of immortalised cancer cells expressing some DCD. Resolution of this issue would require different methodology than that used in the present study.

Other sources suggest that DCD may well be expressed in prostate cancer. In agreement with the data presented above, Wang *et al* (2003) demonstrated DCD/HCAP mRNA expression in prostate cancer cell lines other than PC-3M. However, Wang and coworkers identified DCD/HCAP mRNA in primary prostate cancers and bone metastasis specimens, results that differ from those presented here. One possible explanation for these differences is that Wang *et al* (2003) used RT–PCR rather than real-time PCR.

A further hypothesis on the lack of DCD mRNA expression in the prostate cancer samples analysed in the present study is selective degradation of DCD mRNA. A major control in gene expression is the turnover of mRNA, a tightly regulated process. An important *cis-*acting element controlling the half-life of mRNA are adenylate- and uridylate-rich (AU-rich) elements (AREs) found in the 3′ untranslated regions (UTRs) of many unstable mRNAs. Adenylate-rich elements usually contain repeats of AUUUA, inclusion of AUUUA motifs within 3′UTRs of mRNA may accelerate their decay. Dermcidin has one AUUUA pentamer within its 3′UTR (www.genomatix.de), which may make DCD mRNA more susceptible to degradation. However, for destabilising proteins to form stable complexes with ARE-containing mRNAs many AUUUA pentamers are usually required (Bevilacqua *et al*, 2003).

**Figure 1:**
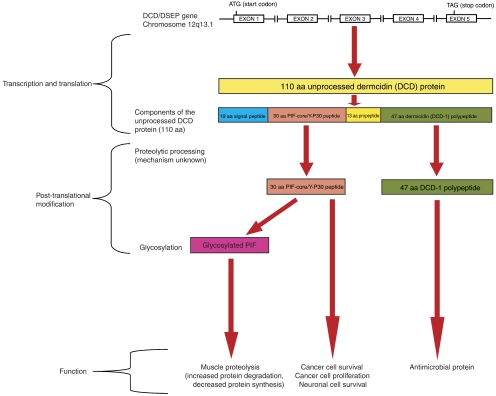
Outline of the dermcidin gene and the processing of its protein products (aa=amino acids). For DCD amino acid sequences see (Lowrie *et al*, 2006). (First appeared in *Curr Opin Clin Nutr Metab Care* Vol **11**(3), pp 208–213 (2008) reproduced with kind permission).

**Figure 2:**
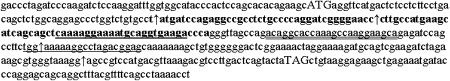
Dermcidin nucleotide sequence. ↑ denotes site of an intron. DCD real-time PCR primer target singly underlined and probe target doubly underlined. PIF-core peptide nucleotide sequence in bold. Start and stop codons are shown in upper case.

Although induction of oxidative stress or hypoxia, two conditions found in the tumour microenvironment, seemed to result in an upregulation of DCD expression in several cancer cell lines, the *C*_T_ values remained in the low/absent range. As such, oxidative stress and hypoxia failed to induce a substantial increase in DCD mRNA expression by the pancreatic or prostatic cell lines studied. However, the Y-P30 peptide subunit of DCD (Figure 1) was originally purified from medium conditioned by human retinoblastoma cells and a mouse hippocampal cell line exposed to hydrogen peroxide (Cunningham *et al*, 1998). It was these findings that stimulated the hypothesis for the present study, that is, that DCD mRNA would be upregulated by the induction of oxidative stress in prostatic and pancreatic cancer cell lines. It is not clear why significant DCD upregulation following exposure to oxidative stress (or hypoxia) did not occur in the present study. However, there are several potential reasons that will form the basis of future studies into DCD. Firstly, tiny concentrations (nanogram per millilitre) of DCD and its protein products have been shown to cause biological effects (Cariuk *et al*, 1997; A Lowrie, unpublished data) and as such improved methods of quantifying accurately small changes in DCD expression caused by environmental stressors, will be essential. Secondly, the stressors used in this study may not have been suitable to cause DCD induction or upregulation. Based on this premise, alternative inducers of oxidative stress and induction of different tumour microenvironmental stresses (e.g., pH imbalance or nutrient deprivation) should be evaluated. Finally, the DCD promoter region may simply not have been induced to transcribe DCD by any of the stimuli used in this study. As such, analysis of the DCD promoter region will be required to progress the study of this interesting gene with pleomorphic biological roles.

Assessment of 58 gastric and oesophageal biopsy samples showed that DCD mRNA expression occurred in a small percentage of gastro-oesophageal malignancy and that expression levels were minimal. Only two gastro-oesophageal cancer RNA samples (4%) expressed moderate DCD mRNA levels. The majority of the gastric and oesophageal cancer patients were weight-losing. Patients with DCD-expressing tumours did not have a significantly greater weight loss, lower BMI or higher CRP than those patients not expressing DCD (data not shown). However, with such low numbers of DCD-expressing tumours, it was difficult to make robust conclusions regarding differences in patient characteristics between groups. Some of the samples utilised in this study had been included in previous work from our laboratory (Deans *et al*, 2006). Deans *et al* (2006), found a higher percentage of PIF-CP mRNA-expressing gastro-oesophageal samples than in the present work. The previous analysis used the MIA-Pa-Ca-2 cell line as a positive control from which to compare relative expression of DCD mRNA. In the present study, MIA-Pa-Ca-2 was found to have a *C*_T_ value of 35.1, equating to borderline moderate DCD expression. As such, we elected not to use MIA-Pa-Ca-2 as a positive control. Thus, PIF-CP expression may have been overestimated in our previous work because of comparison with a control, which has a borderline moderate level of DCD mRNA expression. In the current study, mRNA from PC-3M cells stably transfected with DCD were used as a positive control for DCD real-time PCR. However, as DCD had been artificially overexpressed in the PC-3M cell line this was not an acceptable positive control for use in a relative gene expression method of real-time data expression, as it does not bear any relationship to the *in vivo* situation. Thus *C*_T_ values and DCD/18S rRNA ratios were used in this study to assess DCD mRNA expression.

In the current study, the presence of DCD was assessed using mRNA rather than protein levels. A DCD monoclonal antibody called G-81 is available. However, G-81 recognises the C terminus of DCD-1 rather than full length DCD or PIF-CP/Y-P30 (Minami *et al*, 2004). Polyclonal antibodies to DCD also exist but we have been unable to use these to identify successfully DCD or PIF-CP. PIF is also reported to exist in a glycosylated form and is a putative factor in the increased muscle proteolysis/decreased protein synthesis of cancer cachexia (Lorite *et al*, 1997; Stewart *et al*, 2008). As the existing monoclonal antibody directed against PIF is not specific to glycosylated PIF alone but may recognise a carbohydrate epitope on other molecules, it was not used in the present study (Wieland *et al*, 2007). As mentioned above, more reliable and sensitive methods of quantifying DCD expression are required. In particular a reliable assay for DCD protein estimation is awaited and will be crucial to future studies.

Dermcidin and PIF-CP have been implicated in cancer cell survival in a range of different types of cancer (Porter *et al*, 2003; Lowrie *et al*, 2006; Stewart *et al*, 2007). The results presented here demonstrate that DCD mRNA expression varies both between tumour types and within primary tumours of the same type. There was moderate expression of DCD in one of the pancreatic cell lines but low/absent expression in all prostatic cancer cell lines, findings that correlated with the overall DCD expression patterns by the respective primary cancer tissues. Moreover, in human cell lines where DCD was either not expressed or present in small amounts, microenvironmental stress could not be proven to induce or alter levels of DCD expression. The substantial variability of DCD expression in primary tumours and cell lines and lack of reliable methods of DCD induction *in vitro* may require development of new model systems (perhaps based on the MIA-Pa-Ca-2 cell line) and novel detection methods for the DCD protein itself. These factors make the evaluation of the role of DCD in human tumour biology elusive. As such, the role of DCD expression between different tumour types and within the same tumour type is difficult to ascertain. However, even low levels of DCD expression by a subset of cancer cells may be sufficient to promote clonal cancer cell survival in an adverse environment. All previous studies assessing the various putative roles of DCD and its subunits have involved *in vitro* techniques. However, cell line models have limitations and the true role of DCD *in vivo* remains to be fully elucidated. This study has established that DCD seems to have most relevance to bilio-pancreatic tumours. As such, further studies of DCD expression in human pancreatic cancer and the relationship with patient phenotype and outcome may be the optimum way of taking the biology of human DCD forward.

## Figures and Tables

**Table 1:** Levels of DCD expression in primary tissue and cell lines for each organ site

				**Dermcidin expression level**					
	**Number of samples**				**Number of samples**				
**Organ**	**Benign**	**Cancer**	**Cell line**	**Sample type: benign, cancer or cell line**	**Very high (%)**	**High (%)**	**Moderate (%)**	**Low/absent (%)**	**Not detected/below the limits of detection (%)**
Oesophagus	5^a^	28	0	Benign:	0	0	0	0	5 (100)
				Cancer:	0	0	0	4 (14.3)	24 (85.7)
Stomach	5^a^	20	0	Benign:	0	0	0	0	5 (100)
				Cancer:	0	0	2 (10)	3 (15)	15 (75)
Pancreas	2^b^	5	2	Benign:	0	0	0	0	2 (100)
				Cancer:	0	2 (40)	1 (20)	0	2 (40)
				Cell Line:	0	0	1 (50)	1 (50)	0
Bile duct	0	1	0	Cancer:	0	1 (100)	0	0	0
Prostate	16^c^	52	4	Benign:	0	0	0	0	16 (100)
				Cancer:	0	0	0	0	52 (100)
				Cell Line:	0	0	0	3 (75)	1 (25)
Others	2^d^	1^e^	0	Benign:	0	0	0	0	2 (100)
				Cancer:	0	0	0	1 (100)	0

a Benign samples taken during normal upper gastrointestinal endoscopy examinations.

b Chronic pancreatitis samples.

c Benign prostatic hypertrophy samples.

d Pancreatic liposarcoma and adenoma.

e Pancreatic cystadenoma.

**Table 2:** Details of surgically resected pancreatic tumour pathology in relation to DCD expression level

**Pathology**	**WHO stage**	**DCD *C*_T_ value**	**Level of expression**
Gall bladder metastasis from pancreatic ductal adenocarcinoma	IV	26.7	High
Metastatic pancreatic adenocarcinoma (liver metastasis)	IV	29.3	High
Pancreatic adenocarcinoma (moderately differentiated)	IIa	30.9	Moderate
Cholangiocarcinoma (poorly differentiated adenocarcinoma with perineural and microvascular/ lymphatic invasion)	IVa	29.7	High
Liposarcoma	N/A	35.6	Low/absent

WHO=World Health Organisation; N/A=not applicable.

## References

[bib1] Akerblom IE, Murry LE (1998) Human cachexia associated protein. Incyte Pharmaceuticals Inc.: Palo Alto, CA [628413(5,834,192)]

[bib2] Bevilacqua A, Ceriani MC, Capaccioli S, Nicolin A (2003) Post-transcriptional regulation of gene expression by degradation of messenger RNAs. J Cell Physiol 195: 356–372 12704645 10.1002/jcp.10272

[bib3] Brown JM, Wilson WR (2004) Exploiting tumour hypoxia in cancer treatment. Nat Rev Cancer 4: 437–447 15170446 10.1038/nrc1367

[bib4] Cariuk P, Lorite MJ, Todorov PT, Field WN, Wigmore SJ, Tisdale MJ (1997) Induction of cachexia in mice by a product isolated from the urine of cachectic cancer patients. Br J Cancer 76: 606–613 9303359 10.1038/bjc.1997.433 PMC2228019

[bib5] Cunningham TJ, Hodge L, Speicher D, Reim D, Tyler-Polsz C, Levitt P, Eagleson K, Kennedy S, Wang Y (1998) Identification of a survival-promoting peptide in medium conditioned by oxidatively stressed cell lines of nervous system origin. J Neurosci 18: 7047–7060 9736629 10.1523/JNEUROSCI.18-18-07047.1998 PMC6793258

[bib6] Cunningham TJ, Jing H, Akerblom I, Morgan R, Fisher TS, Neveu M (2002) Identification of the human cDNA for new survival/evasion peptide (DSEP): studies *in vitro* and *in vivo* of overexpression by neural cells. Exp Neurol 177: 32–39 12429208 10.1006/exnr.2002.7979

[bib7] Deans DA, Wigmore SJ, Gilmour H, Tisdale MJ, Fearon KC, Ross JA (2006) Expression of the proteolysis-inducing factor core peptide mRNA is upregulated in both tumour and adjacent normal tissue in gastro-oesophageal malignancy. Br J Cancer 94: 731–736 16495932 10.1038/sj.bjc.6602989 PMC2361198

[bib8] DeWys WD, Begg C, Lavin PT, Band PR, Bennett JM, Bertino JR, Cohen MH, Douglass Jr HO, Engstrom PF, Ezdinli EZ, Horton J, Johnson GJ, Moertel CG, Oken MM, Perlia C, Rosenbaum C, Silverstein MN, Skeel RT, Sponzo RW, Tormey DC (1980) Prognostic effect of weight loss prior to chemotherapy in cancer patients. Eastern Cooperative Oncology Group. Am J Med 69: 491–497 7424938 10.1016/s0149-2918(05)80001-3

[bib9] Lorite MJ, Cariuk P, Tisdale MJ (1997) Induction of muscle protein degradation by a tumour factor. Br J Cancer 76: 1035–1040 9376263 10.1038/bjc.1997.504 PMC2228106

[bib10] Lowrie AG, Wigmore SJ, Wright DJ, Waddell ID, Ross JA (2006) Dermcidin expression in hepatic cells improves survival without N-glycosylation, but requires asparagine residues. Br J Cancer 94: 1663–1671 16685272 10.1038/sj.bjc.6603148 PMC2361319

[bib11] Minami Y, Uede K, Sagawa K, Kimura A, Tsuji T, Furukawa F (2004) Immunohistochemical staining of cutaneous tumours with G-81, a monoclonal antibody to dermcidin. Br J Dermatol 151: 165–169 15270886 10.1111/j.1365-2133.2004.06079.x

[bib12] Nuttall RK, Pennington CJ, Taplin J, Wheal A, Yong VW, Forsyth PA, Edwards DR (2003) Elevated membrane-type matrix metalloproteinases in gliomas revealed by profiling proteases and inhibitors in human cancer cells. Mol Cancer Res 1: 333–345 12651907

[bib13] Porter D, Weremowicz S, Chin K, Seth P, Keshaviah A, Lahti-Domenici J, Bae YK, Monitto CL, Merlos-Suarez A, Chan J, Hulette CM, Richardson A, Morton CC, Marks J, Duyao M, Hruban R, Gabrielson E, Gelman R, Polyak K (2003) A neural survival factor is a candidate oncogene in breast cancer. Proc Natl Acad Sci USA 100: 10931–10936 12953101 10.1073/pnas.1932980100 PMC196905

[bib14] Riddick AC, Barker C, Sheriffs I, Bass R, Ellis V, Sethia KK, Edwards DR, Ball RY (2003) Banking of fresh-frozen prostate tissue: methods, validation and use. BJU Int 91: 315–323 12603403 10.1046/j.1464-410x.2003.03041.x

[bib15] Riddick AC, Shukla CJ, Pennington CJ, Bass R, Nuttall RK, Hogan A, Sethia KK, Ellis V, Collins AT, Maitland NJ, Ball RY, Edwards DR (2005) Identification of degradome components associated with prostate cancer progression by expression analysis of human prostatic tissues. Br J Cancer 92: 2171–2180 15928670 10.1038/sj.bjc.6602630 PMC2361819

[bib16] Schittek B, Hipfel R, Sauer B, Bauer J, Kalbacher H, Stevanovic S, Schirle M, Schroeder K, Blin N, Meier F, Rassner G, Garbe C (2001) Dermcidin: a novel human antibiotic peptide secreted by sweat glands. Nat Immunol 2: 1133–1137 11694882 10.1038/ni732

[bib17] Stewart GD, Lowrie AG, Riddick AC, Fearon KC, Habib FK, Ross JA (2007) Dermcidin expression confers a survival advantage in prostate cancer cells subjected to oxidative stress or hypoxia. Prostate 67: 1308–1317 17626247 10.1002/pros.20618

[bib18] Stewart GD, Skipworth RJ, Ross JA, Fearon KC, Baracos VE (2008) The dermcidin gene in cancer: role in cachexia, carcinogenesis and tumour cell survival. Curr Opin Clin Nutr Metab Care 11: 208–213 18403914 10.1097/MCO.0b013e3282fb7b8d

[bib19] Todorov P, Cariuk P, McDevitt T, Coles B, Fearon K, Tisdale M (1996) Characterization of a cancer cachectic factor. Nature 379: 739–742 8602222 10.1038/379739a0

[bib20] Valko M, Rhodes CJ, Moncol J, Izakovic M, Mazur M (2006) Free radicals, metals and antioxidants in oxidative stress-induced cancer. Chem Biol Interact 160: 1–40 16430879 10.1016/j.cbi.2005.12.009

[bib21] Vaupel P, Hockel M, Mayer A (2007) Detection and characterization of tumor hypoxia using pO2 histography. Antioxid Redox Signal 9: 1221–1235 17536958 10.1089/ars.2007.1628

[bib22] Wang Z, Corey E, Hass GM, Higano CS, True LD, Wallace Jr D, Tisdale MJ, Vessella RL (2003) Expression of the human cachexia-associated protein (HCAP) in prostate cancer and in a prostate cancer animal model of cachexia. Int J Cancer 105: 123–129 12672042 10.1002/ijc.11035

[bib23] Wieland BM, Stewart GD, Skipworth RJ, Sangster K, Fearon KC, Ross JA, Reiman TJ, Easaw J, Mourtzakis M, Kumar V, Pak BJ, Calder K, Filippatos G, Kremastinos DT, Palcic M, Baracos VE (2007) Is there a human homologue to the murine proteolysis-inducing factor? Clin Cancer Res 13: 4984–4992 17785548 10.1158/1078-0432.CCR-07-0946

